# The impact of the COVID-19 pandemic on medical student mental health

**DOI:** 10.1371/journal.pmen.0000264

**Published:** 2025-04-08

**Authors:** Julie H. Wolfe, Stephanie Lehto, Joseph T. Sakai, Susan K. Mikulich-Gilbertson, Rachel A. Davis

**Affiliations:** 1 Department of Psychiatry, University of Colorado School of Medicine, Aurora, Colorado, United States of America; 2 Department of Biostatistics & Informatics, University of Colorado School of Public Health, Aurora, Colorado, United States of America; 3 Department of Neurosurgery, University of Colorado School of Medicine, Aurora, Colorado, United States of America; University of Santo Tomas College of Science, PHILIPPINES

## Abstract

**Objectives:**

The authors studied the impact that the COVID-19 pandemic had on medical students’ mental health.

**Methods:**

US medical students received an online survey with questions from standardized rating scales in 2019 and 2021 in the fall prior to matriculation and in the spring of each year in medical school. The authors compared reports of depression, anxiety, stress, obsessive compulsive symptoms, substance use, impostor phenomenon, maladaptive perfectionism and childhood trauma symptoms in medical students pre-pandemic (2019) and during pandemic (2021) across all four years of medical school.

**Results:**

For depression, anxiety, binge drinking, non-cannabis substance use and Impostor Phenomenon, there were significant differences among medical school classes. For many measures, ratings were lowest at pre-matriculation, rose during medical school, and declined in the fourth year. When assessing pre-pandemic vs during pandemic ratings, 2021 scores were significantly higher than in 2019 for depression, generalized anxiety, and Impostor Phenomenon. Binge drinking and non-cannabis use were significantly lower in 2021 than in 2019. There were not significant differences for Depression, Anxiety and Stress Scale anxiety (“DASS anxiety), obsessive compulsive symptoms, and adverse childhood events.

**Conclusion:**

Medical school and the COVID-19 pandemic were associated with worsening mental health. During the COVID-19 pandemic, many of the measures of mental health worsened, and the pattern across years remained fairly consistent.

## Introduction

Prior work has identified medical students as a group at higher risk of poor mental health outcomes. For example, medical students begin medical school with mental health profiles similar to controls [1] but, during the course of medical school, develop higher rates of depression and anxiety [2]. Although the bulk of available work with medical students has focused on depression and broad definitions of anxiety, some work has also investigated other domains, such as obsessive compulsive disorder [3], impostor phenomenon [4], perfectionism [4], substance use [5] and adverse childhood experiences [6].

In March of 2020 the COVID pandemic led to dramatic changes worldwide. Specifically, medical students experienced many curricular changes and new challenges related to quarantine and other effects of COVID, including a rapid shift to virtual lectures, removal of students from clinical rotations, uncertainty with USMLE testing, a move to virtual residency interviews, and the elimination of away rotations [7–9].

It is not surprising that these changes had effects on medical students’ mental health. A small but growing body of work supports that medical students experienced higher stress [10], depression [11], anxiety [12], substance use [11] and increased use of mental health resources [11] during the pandemic. However, the existing literature has several methodological limitations. Most studies lack comparable pre-pandemic data [7,10,11], used convenience sampling (e.g., recruiting participants through social media and asking medical school leaders and coordinators to distribute the survey to students) [7], or examined only pre-clinical students [8] or students on a specific clinical rotation [13]. To our knowledge, there are no studies comparing medical school classes (pre-matriculation through fourth year) assessed just prior to and following the implementation of the COVID-related lockdown.

This paper seeks to add to the existing literature on the effects of the pandemic on medical students’ mental health. We report on a sample from a single medical school with students (including those just matriculating and first through fourth year students) assessed in 2019 just prior to the pandemic and in 2021 early in the pandemic using a broad array of mental health measures. We tested differences in mental health measures by comparing students assessed pre vs during pandemic (2019 vs 2021) and by medical school class (e.g., pre-matriculation through fourth year).

## Methods

### Study design

This data is part of an ongoing study at a US LCME-accredited medical school, which started recruiting participants beginning on May 9, 2018 with active enrollment at the time of publication. Medical students completed a voluntary, online survey in the fall prior to matriculation (“PM”; i.e., the fall of 2019 and 2021) and in the spring of each of the four years (“MS1”, “MS2”, “MS3”, “MS4”; i.e., the spring of 2019 and 2021). The data represents two cross-sectional samples from medical school classes (PM, MS1, MS2, MS3, MS4) surveyed in 2019 (“pre-pandemic”) and 2021 (“during pandemic”). Participants received postcard informed consent prior to starting the rating scales, and continuing the survey confirmed consent. All responses were anonymous, and no questions required responses with identifiable information. Students were given the opportunity to fill out an optional survey to request to be contacted by faculty to schedule an appointment in the Student and Resident Mental Health clinic located on the medical campus. The responses from the optional survey were not linked to the primary survey and those data are not in this report. The study was approved by the institutional review board.

### Survey measurements

The survey included questions from standardized rating scales to evaluate symptoms of depression, anxiety, stress, OCD, perfectionism, impostor phenomenon, adverse childhood experiences, and screening questions about substance use.

### Patient health questionnaire-9 (PHQ-9) – depression

The PHQ-9 includes 9 questions to assess for depressive symptoms [14]. Students were asked to rate their symptoms over the past 2 weeks. Each item was scored on a four-point scale (0–3 points). The total score was the sum of the scores on all items.

### Depression, anxiety and stress scale (DASS-21) – depression, anxiety, stress

The DASS-21 includes 7 questions in each of the 3 sub-groups (depression, anxiety, stress; for a total of 21 questions) to measure for the presence of these symptoms [15]. Each item was scored on a four-point scale (0–3 points). The sum of each of the questions in the 3 sub-groups was then multiplied by 2 to calculate the final score.

### Generalized anxiety disorder-7 (GAD-7) - anxiety

The GAD-7 includes 7 questions to assess for anxiety symptoms [16]. Students were asked to rate their symptoms over the past 2 weeks. Each item was scored on a four-point scale (0–3 points). The total score was the sum of the scores on all items.

### Clance IP scale (CIPS) – impostor phenomenon

The CIPS includes 20 questions to assess the presence of impostor phenomenon characteristics [17]. Each question was scored on a 5-point scale (1–5). The total score was the sum of the scores on all items. The standard scoring algorithm was used to create a categorial variable of having impostor characteristics. We used a cut-off score of 40 which corresponds to moderate impostor characteristics to obtain frequency of students that were positive for moderate IP or greater.

### Substance use

Three one-question screeners assessed substance use [18]. Single-item screening tools for hazardous drinking and alcohol use disorder have been found to perform nearly at the same level as the AUDIT-C, a validated screening tool to identify hazardous drinking [19,20].

“How many times in the past year have you had X or more drinks in a day?”

(where X is 5 for men and 4 for women), hereto referred to as binge drinking.

“How many times in the past year have you used an illegal drug (excluding cannabis) or used a prescription medication for non-medical reasons?”

“How many times in the past year have you used cannabis?”

For these analyses, we dichotomized the results into yes or no (e.g., one or more episodes of binge drinking in the prior year was counted as “yes”).

### Obsessive-compulsive inventory (OCI-R) – obsessive compulsive symptoms

The OCI-R includes 18 questions to assess for the presence of OCD symptoms [21]. Each item was scored on a 5-point scale (0–4 points). The total score was the sum of the scores on all items.

### Adverse childhood experiences questionnaire (ACE-Q) – childhood trauma symptoms

The ACE-Q includes 10 questions to measure childhood trauma. Each question was scored as 0 or 1. The total score was the sum of the scores on all items. We used a cut-off score of 4 to create our categorical variable of significant adverse childhood experiences [22].

### Almost perfect scale-revised (APS-R) – adaptive vs maladaptive perfectionism

The APS-R includes 23 questions to distinguish between adaptive and maladaptive perfectionism [23]. Each item was scored on a 7-point scale (1–7 points). The total score was the sum of the scores on all items. The standard scoring algorithm was used with a cut-off score of 42 for the standards subscale to create a categorical variable of perfectionist. Then we used a cut-off score of 42 for the discrepancy subscale to identify maladaptive perfectionism.

### Hypotheses

We hypothesized *a priori* that the pre-pandemic (2019) versus during pandemic (2021) outcomes would be worse during the pandemic for depression, anxiety/stress, OCD and substance use. We hypothesized *a priori* that pre-pandemic (2019) versus during pandemic (2021) outcomes would not be different for maladaptive perfectionism, impostor phenomenon, and adverse childhood experiences.

### Data analysis

Medical student characteristics were compared between 2019 and 2021 cohorts with chi-square tests. All quantitative outcome measures were square root transformed to provide approximately normal distributions for parametric testing (i.e., analysis of variance ANOVA). Mean estimates were back-transformed to the original scale for presentation in the figures. We conducted ANOVAs or multiple logistic regressions for each outcome as appropriate to evaluate the effects of calendar year (2019 vs. 2021), medical school class (PM, MS1, MS2, MS3, MS4) and their interaction. Non-significant interactions (p > 0.10) were removed, and the models were re-run for those outcomes. Significance level was set at alpha = 0.05 two-tailed, except for interaction terms more liberally alpha = 0.10. Analyses were performed using SPSS and SAS.

## Results

Of the 1,819 students that were invited to participate in the study, 606 answered at least one survey question (33% response rate). Table 1 describes demographics and student loan burden for students surveyed in 2019 and separately 2021.

**Table 1:** Medical Student Characteristics in 2019 vs. 2021; number (% of non-missing responses).

	2019	2021	Statistic (df); p-value
***Age in years (missing n=61)***			X^2^(3) = 2.06, p=0.56
18-21	3 (0.9%)	3 (1.6%)	
22-25	173 (49.1%)	88 (45.6%)	
26-29	129 (36.6%)	69 (35.8%)	
30+	47 (13.4%)	33 (17.1%)	
***Pronouns (missing n=61)***			X^2^(3) = 7.73, p=0.052
He/Him	150 (42.6%)	61 (31.6%)	
She/Hers	198 (56.3%)	129 (66.8%)	
They/Their	1 (0.3%)	0 (0.0%)	
Prefer not to answer	3 (0.9%)	3 (1.6%)	
***Ethnicity (missing n=62)***			X^2^(1) = 1.26, p=0.26
Minority	122 (34.8%)	58 (30.1%)	
Non-Minority	229 (65.2%)	135 (69.9%)	
***Current amount of outstanding student loans (missing n=61)***			X^2^(2) = 10.2, p=0.006
No student loans	106 (30.1%)	84 (43.5%)	
<$150K	156 (44.3%)	65 (33.7%)	
$150K and greater	90 (25.6%)	44 (22.8%)	

The figures associated with each outcome listed below illustrate the least square means (back transformed to original scale) or percentages endorsing as appropriate for each subgroup (medical school class by year) based on the full model containing year, medical school class, and their interactions.

### Depressive symptoms (Fig 1A and 1B)

Neither depression measure had a significant year by class interaction. PHQ-9 score was significantly higher in 2021 compared to 2019 (F(1,530) = 9.5; p=0.002) and differed across medical school classes (F(4,530) = 15.7; p<0.0001) with the MS2 classes having the highest scores and PM classes having the lowest scores on average. DASS Depression score was significantly higher in 2021 compared to 2019 (F(1,505) = 4.1; p=0.04) and differed significantly across medical school classes (F(4,505) = 10.0; p<0.0001) with the MS2 classes having higher scores in both years and MS1 class having an even higher score in 2021 than for other medical school classes. As with PHQ-9, the PM classes had the lowest scores on average for DASS Depression.

**Fig 1:**
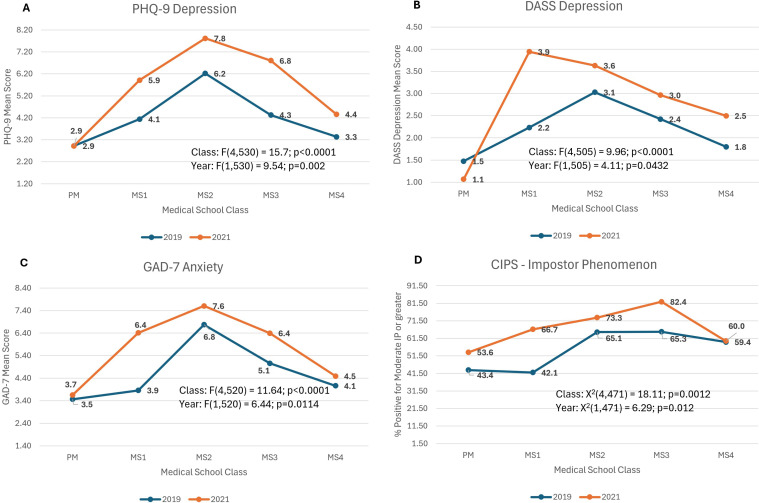
Models in which mental health was worse in 2021 vs 2019. ***PHQ-9:*** mean score of Patient Health Questionnaire-9 to assess for depression, ***DASS-Depression:*** Depression, Anxiety, Stress-21 Scale; mean score of Depression sub-group, ***CIPS:*** Clance Impostor Phenomenon Scale to assess for impostor phenomenon characteristics; % of respondents that met criteria for Moderate Impostor Phenomenon or greater, ***Class*:** year in medical school; PM (pre-matriculation), MS1 (1^st^ year medical student), MS3 (3^rd^ year medical student), MS4 (4^th^ year medical student).

### Generalized anxiety symptoms (Fig 1C)

The GAD-7 score did not have a significant year by class interaction but was significantly higher in 2021 compared to 2019 (F(1,520) = 6.4; p=0.01) and differed across medical school classes (F(4,520) = 11.6; p<0.0001) with the MS2 classes having the highest scores and PM classes having the lowest scores.

### Impostor phenomenon (Fig 1D)

CIPS score showed no significant year by class interaction but was significantly higher in 2021 when compared to 2019 (X^2^(1) = 6.3; p=0.01) and differed across medical school classes (X^2^(4) = 18.1; p=0.001). For both years, the MS4 classes had the highest scores and the PM classes had the lowest scores.

### Binge drinking (Fig 2A)

Reports of one or more episodes of binge drinking in the past year showed no year by class interaction and did not differ across medical school classes but was significantly lower in 2021 when compared to 2019 (X^2^(1) = 7.9; p=0.005)).

**Fig 2:**
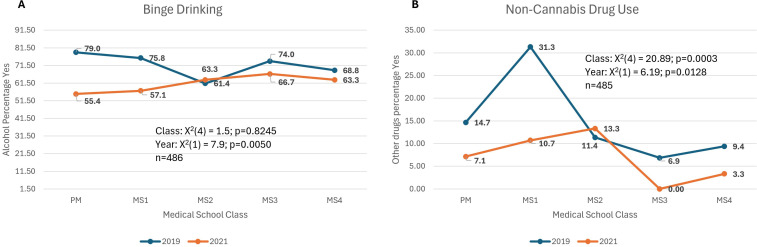
Models in which mental health was worse 2019 vs 2021. ***Binge Drinking*:** % of respondents that answered yes to a 3-question screener “How many times in the past year have you had X or more drinks in a day?” (X is 5 for men and 4 for women), ***Non-Cannabis Use:*** % of respondents that answered positive (≥1 episodes) to “How many times in the past year have you used an illegal drug (excluding cannabis) or used a prescription medication for non-medical reasons?”, ***Year:*** the year survey was conducted; 2019 or 2021.

### Non-cannabis drug use (Fig 2B)

Other drug (non-cannabis) use in the past year showed no year by class interaction. It was significantly different lower in 2021 when compared to 2019 (X^2^(1) = 6.2; p=0.012) and across medical school classes (X^2^(4) = 20.9; p=0.0003) such that on average the MS1 class had the highest rate and the MS3 class had the lowest rate of ever using in the past year.

### DASS - anxiety symptoms (Fig 3A)

The DASS-anxiety score did not have a significant year by class interaction. It was not significantly different by year, but differed significantly across medical school classes (F(4,511) = 5.2; p=0.0004) with anxiety scores peaking in the MS2 classes.

**Fig 3:**
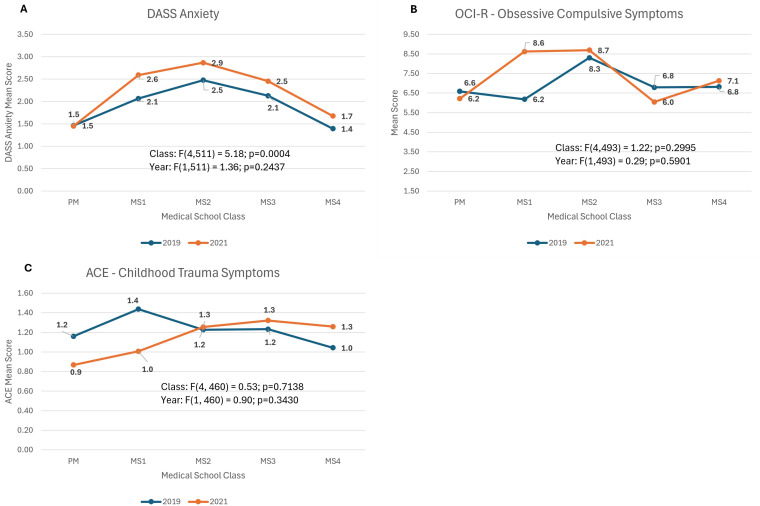
Models in which there was no difference between mental health scores in 2021 vs 2019. ***DASS-Anxiety:*** Depression, Anxiety, Stress-21 Scale; mean score of Anxiety sub-group, ***OCI-R:*** mean of Obsessive-Compulsive Inventory to screen for Obsessive Compulsive symptoms, ***ACE:*** mean of Adverse Childhood Experiences Questionnaire to assess for childhood trauma symptoms.

### Obsessive compulsive symptoms (Fig 3B)

OCI-R scores showed no year by class interaction nor main effects of year nor medical school class.

### Childhood trauma symptoms (Fig 3C)

ACEs showed no year by class interaction nor main effects of year nor medical school class.

### Stress symptoms (Fig 4A)

The DASS-Stress score had a significant year by class interaction (F(4,505) = 2.1; p=0.08) with 2021 higher for each class when compared to 2019 but especially for the MS1 and MS2 classes.

**Fig 4:**
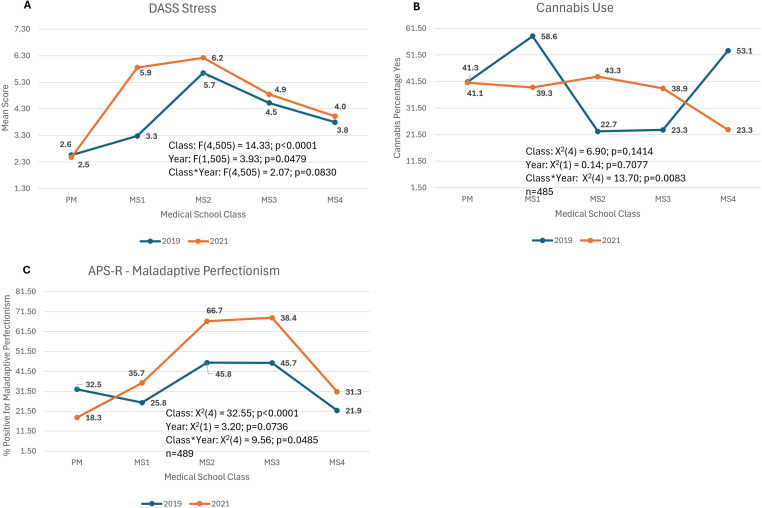
Models in which the class by year interaction was significant. ***DASS-Stress:*** Depression, Anxiety, Stress-21 Scale; mean score of Stress sub-group, ***Cannabis Use:*** % of respondents that answered positive (≥1 episodes) to “How many times in the past year have you used cannabis?”,***APS-****R*: Almost Perfect Scale Revised to distinguish between adaptive and maladaptive perfectionism; % of respondents that met criteria for maladaptive perfectionism*.*

### Cannabis Use (Fig 4B)

Cannabis use in the past year had a significant year by class interaction (X^2^(4) = 13.7; p=0.01) with 2019 higher than 2021 for PM, MS1 and MS4 classes and lower than 2021 for MS2 and MS3 classes.

### Almost perfect scale/maladaptive perfectionism (Fig 4C)

The APS had a significant year by class interaction (X^2^(4) = 9.6; p=0.05) with lower maladaptive perfectionism in 2021 than in 2019 in PM and higher maladaptive perfectionism in 2021 than in 2019 for MS1-MS4 classes.

## Discussion

This study examined two cohorts of medical students assessed just prior to and early in the COVID pandemic using a broad range of potential mental health measures. Our study found that: (1) medical school was associated with worsening mental health in a predictable pattern; (2) the COVID-19 pandemic was associated with worsening on many of our measures of mental health, and the pattern across medical school years (see first finding) remained fairly consistent (e.g., the curve was simply pushed upward but retained its shape); and (3) in some instances, the pandemic was related to lower prevalence of some, presumably, negative behaviors such as binge drinking.

Medical school was associated with worsening mental health in a predictable pattern. Looking across the classes of medical school in 2019, we see a predictable pattern with lowest rates at pre-matriculation, rising rates across MS1-MS3 classes, and a return to lower levels at the end of medical school (see Fig 1A-1D). This pattern is consistent with prior work showing higher rates of depression and anxiety among medical trainees, including medical students, residents, and fellows with five to eight times higher rates of depression and anxiety when compared to age-matched US population samples [2]. This suggests that factors intrinsic to medical school contribute to the worsening of mental health [24]. Given this pattern, a focus on curriculum innovations for the MS2 and MS3 classes may help mitigate some of these factors.

*The COVID-19 pandemic was associated with worsening of many of the measures of mental health, and the pattern across years remained fairly consistent*. For many of the measures, students at pre-matriculation in 2019 and 2021 had similar scores, despite the pandemic. Across medical school classes, the shape of the curve (see previous paragraph) remained similar in 2021 compared to 2019 but was shifted upward. It appears that the impact of the COVID pandemic and medical school together may have contributed to substantial worsening of mental health for many students. These findings are consistent with another study that found a significant decrease in pre-pandemic versus pandemic wellness, even when controlling for medical school class [7]. Many of the life changes that occurred because of the COVID pandemic likely contributed. Medical students experienced a lower sense of purpose as they were labeled as non-essential and removed from the frontlines, especially early in the pandemic [7]. There were difficulties with scheduling board exams, and many important events such as graduation and match day were cancelled due to social distancing restrictions [7]. As classes shifted to virtual, students spent more time at home with less social interactions [7] and felt less connected to their medical school and their classmates [8]. Thus, fostering community, social connectedness, a sense of belonging and meaning/purpose within the medical school community for students might be an important strategy to mitigate mental health concerns seen during the MS2–3 classes.

*The pandemic was related to lower prevalence of some, presumably, negative behaviors such as substance use.* Both binge drinking and non-cannabis drug use were lower during the pandemic. Possible contributing factors for the decline may be related to a lack of social interaction due to social distancing limitations, possible changes in student’s living environments and less availability of non-cannabis substance use. Recent work supports a general increase in alcohol use during the pandemic among US adults in terms of drinking days [25] and consumption [26]. However, the pandemic impact on binge drinking in young adults is mixed with studies showing both increases and decreases in binge drinking behaviors [27,28]. For medical students specifically, we hypothesize that a reduction of in-person social interactions may be associated with the reported decrease in binge drinking in 2021. This is consistent with prior work linking social interactions with heavier drinking [29]. Our measure of binge drinking is yes/no based on having at least a single occurrence over the past year and thus limited. We have no information on the frequency of binge drinking in this sample [30].

Some measures did not differ in pre-COVID and during COVID comparisons. For example, adverse childhood experiences (ACE) scores were similar across years, consistent with our *a priori* hypotheses (given that the questions relate to experiences before age 18 we would not expect differences between groups). Although on the surface, such a result might seem simple and expected, it does provide some reassurance that the stress of the pandemic did not lead to over-reporting. For other measures, the outcomes contradicted our *a priori* hypotheses. For example, scores on obsessive compulsive symptoms remained similar between years despite the increased stress in 2021 (see DASS Stress results).

In summary, the results from this study contribute to the broader literature on medical student mental health generally and the COVID-19 pandemic’s impact on mental health specifically, but our results should be viewed within the context of a few limitations. First, our response rates raise concerns about potential bias, which should be carefully considered when interpreting our results. However, the available literature in this area suffers from the same concern, with some studies having much lower response rates (e.g., 10–12% [9,12]) or being unable to determine response rates [7,11], or conducting online recruitment via social media [7,11]. Thus, within the context of that broader literature, our results add meaningful additional data. Second, this study surveyed students from a single medical school, which may limit generalizability to the broader medical school population; some of the experiences of our students may be idiosyncratic. However, we are reassured that the patterns we observed pre-COVID are in line with the existing literature. Third, the rating scales used in our study asked about functioning over the past 2–4 weeks, which may not have been representative of a participant’s experience over the past year. Lastly because of scheduling, we were not able to have participants complete the surveys on the exact same day across all four classes which may have introduced some variability into the results. Our study also has several strengths. Most notably, this study is unique in collecting the same measures prior to and following the onset of the pandemic. In addition, we present data from students just prior to matriculation and across all four medical school years (some prior studies limited their work to pre-clinical or clinical years), allowing visualization of patterns across medical school years prior to and during the pandemic.

### Implications

This work highlights the need for innovative changes to combat mental concerns during the medical school years. Most notably, studies to further identify specific time periods during medical school where mental health may be affected could better allow for more targeted interventions. Specific emphasis on changes could be placed on the MS2-MS3 years. Additionally, research involving multi-site data collection could increase the sample size and offer further insight into the mental health of medical students across geographic locations to better identify characteristics of medical students and medical school curricula that may contribute to risk. Our hope is that this information will ultimately allow for improved access to timely and effective health care during medical school and beyond.
